# The Impact of the COVID-19 Pandemic on Coronary Interventional Cardiology Activity in King Abdulaziz Medical City: A Retrospective Study

**DOI:** 10.7759/cureus.36453

**Published:** 2023-03-21

**Authors:** Nouf Z AlBattal, Malak N AlShebel, Mohammed Balgaith, Hatoon M Alghuson, Lama A AlShenaifi, Reema A Ghamdi

**Affiliations:** 1 College of Medicine, King Saud Bin Abdulaziz University for Health Sciences, Riyadh, SAU; 2 Department of Research, King Abdullah International Medical Research Center, Riyadh, SAU; 3 Department of Interventional Cardiology, King Abdulaziz Medical City, Riyadh, SAU

**Keywords:** nstemi, stemi, pci, covid-19, interventional cardiology

## Abstract

Background: The COVID-19 pandemic has decreased the number of patients undergoing coronary interventional procedures. This study aimed to identify the impact of the COVID-19 pandemic on the volume of patients and the types of interventional cardiology procedures performed at King Abdulaziz Cardiac Center (KACC) in Riyadh, Saudi Arabia.

Methods: A retrospective chart review was undertaken with a sample size of 301 patients aged over 18 years, who underwent various cardiac interventions at King Abdulaziz Cardiac Center (KACC) between March 15, 2019, and February 29, 2020 (prior to the pandemic, group A), and between March 1, 2020, and March 15, 2021 (during the pandemic, group B). The BESTCare 2.0 system (ezCaretech, Seoul, South Korea) was used to collect data, Microsoft Office Excel (Microsoft^®^ Corp., Redmond, WA) was utilized for data entry, and the Statistical Package for Social Sciences software (IBM SPSS Statistics, Armonk, NY) was employed for data analysis.

Results: There was a 21.4% decrease in the number of procedures performed during the pandemic. The largest age group within the population was ≥60 years, comprising 43.5% and 52.3% of groups A and B, respectively. Most patients had a body mass index (BMI) of >30, i.e., 43.5% of patients before the pandemic and 47.7% after the pandemic. In group A, 39.9% were smokers and 60.6% in group B. The prevalence of hypertension and obesity was higher in group B, i.e., 77.3% and 42.3%, respectively. The incidence of ST-elevation myocardial infarction (STEMI) was 39.9% in group A and 39.4% in group B. For non-ST-elevation myocardial infarction (NSTEMI), the comparable statistics were 56.5% and 49.2%, respectively. In groups A and B, readmission frequencies were 17.9% and 20%, respectively.

Conclusion: The study indicates a minor decline in the number of percutaneous coronary interventions (PCIs) conducted in the interventional cardiology department of King Abdulaziz Cardiac Center (KACC) immediately following the COVID-19 outbreak, reflecting a steady activity in the center.

## Introduction

The first percutaneous transluminal angioplasty was performed on a patient in 1977. Since this date, interventional cardiology has improved more rapidly than any other form of treatment within the specialty of cardiovascular disease (CVD) [1]. According to the Society for Cardiovascular Angiography and Interventions, interventional cardiology is defined as “the specialized branch of cardiology that treats coronary artery disease with balloon angioplasty and stenting therapies that unblock clogged arteries that supply blood to the heart, stop heart attacks, and relieve angina or chest pain” [2]. However, the current circumstances arising from the novel coronavirus outbreak have led to a significant global setback in healthcare, including in interventional cardiology units [3].

CVD is the leading cause of morbidity and mortality worldwide. In the Kingdom of Saudi Arabia (KSA), for instance, coronary artery disease (CAD) has a prevalence of 5.5%, which is alarming considering that the prevalence of risk factors for CAD is increasing. Further interventional measures are required in order to prevent CAD from developing into an epidemic, for instance, a national prevention program offered for high-risk populations to reduce modifiable risks [4]. Although the rate of percutaneous coronary intervention (PCI) with ST-elevation myocardial infarction (STEMI) in Saudi Arabia has increased significantly in the previous years, the rate of PCIs (23 per 1,000,000) was compared to the rate of thrombolytic therapy (161 per 1,000,000) in 2011, which might be attributed to the presence of only four hospitals with 24/7 primary PCI service across the country [5].

The globe is undergoing a massive crisis owing to the ongoing pandemic of COVID-19 disease. This pandemic has modified how healthcare systems function worldwide. The KSA was one of the quickest countries to recognize the size of the threat and implement appropriate measures to limit the spread of the virus. The Ministry of Health (MOH) surveys all healthcare practices and services in the KSA and has delivered excellent preventive, curative, and rehabilitative healthcare services during the crisis. This has been achieved by instigating public-private partnership (PPP) healthcare models and by facilitating access to readily available testing centers to reduce the outbreak. Digital health was instigated for many crucial services, e.g., the “My Health” application, which enabled the provision of remote medical treatment and prescriptions [6].

In conclusion, to achieve a better understanding of the overall situation, the need for a study investigating these aspects in the authors’ institution has been highlighted. Therefore, the current research aims to assess the volume and impact of COVID-19 on the interventional cardiology department at the King Abdulaziz Cardiac Center (KACC), with a focus on in-patients presenting with acute coronary syndromes (ACS).

## Materials and methods

A retrospective chart review design was chosen in order to determine whether the volume of in-patients and the number and type of interventional procedures performed had changed during the COVID-19 pandemic. This study was conducted at King Abdulaziz Cardiac Center (KACC), King Abdulaziz Medical City (KAMC), Ministry of National Guard Health Affairs, Riyadh, Kingdom of Saudi Arabia (KSA). This National Guard hospital is a 900-bed tertiary care facility that provides healthcare services to approximately 600,000 KSA National Guard soldiers, employees, and their families. The KACC has 150 beds, 45 of which are intensive care unit (ICU) beds; these include a medical cardiac ICU, cardiac surgical ICU, pediatric cardiac ICU, and coronary care unit. Various cardiac services such as echocardiography, transesophageal echocardiogram (TEE), computed tomography (CT) coronary angiogram, electrocardiography services, and catheterization laboratory are offered in the center; many new technologies are used, thus improving the care [7].

A non-probability, consecutive sampling technique was used to collect data, including patient demographic characteristics, interventions, and lengths of stay. The investigators, after attaining the IRB approval (SP21R-162-05) from King Abdullah International Medical Research Center (KAIMRC), gathered data from March 2019 to March 2021 from the KACC KAMC computer system, BESTCare 2.0 system (ezCaretech, Seoul, South Korea), and the picture archiving and communication system to identify the changes in the volume of patients between March 15, 2019, and March 15, 2021. Patients were selected based on their interventions; the same inclusion criteria included male and female Saudi and non-Saudi in-patients over 18 years who had presented with acute coronary syndromes (ACS) and/or undergone one or more percutaneous coronary interventions (PCIs). The studied variables included patients’ demographic characteristics, i.e., age, gender, body mass index (BMI), risk factors, past medical and surgical history, hypertension, diabetes, smoking, family history, weight, dyslipidemia, length of stay, and type of intervention. In order to attain a 95% confidence level and a margin of error of 5%, a sample size of 301 patients was calculated for this study using the Raosoft (Raosoft, Inc., Seattle, WA) sample size calculator, given a population of 1375 patients.

Data were collected using an Excel (Microsoft® Corp., Redmond, WA) worksheet and separated into two groups. The first cohort, group A, included patients admitted between March 15, 2019, and February 29, 2020, i.e., prior to the pandemic; the second group, group B, comprised patients admitted between March 1, 2020, and March 15, 2021, i.e., during the pandemic.

Hypertension was defined as systolic blood pressure (BP) of ≥140 mmHg or diastolic BP of ≥90 mmHg or the use of antihypertensive medication [8]. Diabetes was defined as glycosylated hemoglobin (HbA1c) of ≥6.5%, fasting blood sugar of ≥126 mg/dL, two-hour blood sugar of ≥200 mg/dL, random blood sugar of ≥200 mg/dL, or the use of oral hypoglycemic agents [9]. Dyslipidemia was defined as total cholesterol level of ≥240 mg/dL, triglyceride level of ≥150 mg/dL, low-density lipoprotein cholesterol level of ≥140 mg/dL, high-density lipoprotein cholesterol level of <40 mg/dL, or the use of a lipid-lowering drug [10].

After the data collection, the variables were imported into the Statistical Package for Social Sciences version 28.0 (IBM SPSS Statistics, Armonk, NY) for analysis. The type of intervention and the demographic characteristics described above were analyzed and reported as frequency and percentages. Numerical variables were presented as mean ± standard deviation. A t-test was used to measure any association between numerical variables, e.g., the number of patients and procedures before and after the COVID-19 period; the outcome was presented as mean differences. A p-value of <0.05 was considered to be statistically significant. Any missing data were reported and explained.

Confidentiality and anonymity were ensured for all patient data, as no information relating to personal identification was used. Measures to protect participants’ privacy were taken, including the use of computer passwords, firewalls, antivirus software, and encryption; these protected data from unauthorized individuals, loss, theft, or modification.

## Results

**Figure 1:**
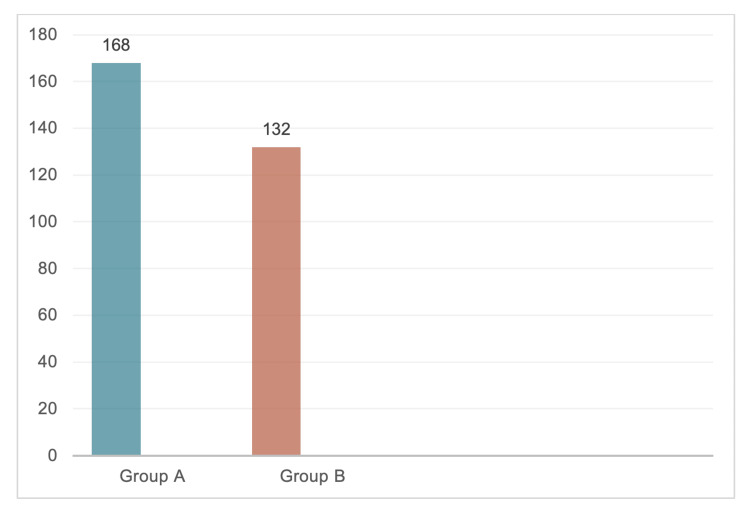
Number of total PCI done in both groups There was a 21.4% decrease in the number of procedures during the pandemic (group B) PCI: percutaneous coronary intervention

Figure 1 shows the sample sizes of groups A (patients admitted before the pandemic) and B (patients admitted during the pandemic), which were 168 and 132, respectively.

Table 1 presents the sociodemographic profile of the population. Most patients were ≥60 years of age; patients in this age range comprised 43.5% and 52.3% of groups A and B, respectively. In group A, the proportion of males was 78.6% and 21.4% in females: the equivalent percentages in group B were 84.1% and 15.9%, respectively. Patients of Saudi nationality made up 83.9% of group A and 92.4% of group B; 30% of patients had a body mass index (BMI) of ≥30 in group A; 47.7% had a BMI of ≥30 in group B; 39.9% and 60.6% of patients were smokers in groups A and B, respectively.

**Table 1:** Sociodemographic profile of the patients BMI: body mass index

Demographical characteristics		Group A (N = 168)		Group B (N = 132)	
		N	%	N	%
Age	30-40 years	11	6.5	10	7.6
	40-50 years	32	19.0	15	11.4
	50-60 years	52	31.0	38	28.8
	≥60 years	73	43.5	69	52.3
Gender	Male	132	78.6	111	84.1
	Female	36	21.4	21	15.9
Nationality	Saudi	141	83.9	122	92.4
	Non-Saudi	27	16.1	10	7.6
BMI	<18.5	2	1.2	1	0.8
	18.5-24.9	33	19.6	20	15.2
	25-29.9	60	35.7	48	36.4
	≥30	73	43.5	63	47.7
Smoker	No	101	60.1	52	39.4
	Yes	67	39.9	80	60.6
Surgical history	No	101	60.1	110	83.3
	Yes	67	39.9	22	16.7
Past medical history	No	77	45.8	97	73.5
	Yes	91	54.2	35	26.5
Family history	No	155	92.3	123	93.2
	Yes	13	7.7	9	6.8
Hypertension	No	57	33.9	30	22.7
	Yes	111	66.1	102	77.3
Dyslipidemia	No	59	35.1	51	38.6
	Yes	109	64.9	81	61.4
Chronic kidney disease	No	138	82.1	107	81.1
	Yes	30	17.9	25	18.9
History of coronary heart disease	No	83	49.4	41	31.1
	Yes	85	50.6	91	68.9
Hypothyroidism	No	157	93.5	123	93.2
	Yes	11	6.5	9	6.8
Congestive heart disease	No	154	91.7	122	92.4
	Yes	14	8.3	10	7.6
Atrial fibrillation	No	158	94.0	124	93.9
	Yes	10	6.0	8	6.1

The results shown in Table 2 indicate the incidence of ST-elevation myocardial infarction (STEMI) to be 39.9% in group A and 39.4% in group B. No difference was seen in the relative percentages of STEMI patients before and after the pandemic.

**Table 2:** Number of STEMI patients in group A and group B STEMI: ST-elevation myocardial infarction

STEMI		Group A		Group B	
		Frequency	%	Frequency	%
No		101	60.1	80	60.6
Yes		67	39.9	52	39.4
STEMI	N	Mean	Standard deviation	Independent t-test (p-value)	
Group A	168	0.399	0.490	0.085 (0.932)	
Group B	132	0.394	0.490		

Table 3 shows the incidence of patients presenting with non-ST-elevation myocardial infarction (NSTEMI), i.e., 59,5% in group A and 49.2% in group B. Again, no difference in the relative proportions of patients presenting with NSTEMI was identified between the studied periods.

**Table 3:** Number of NSTEMI patients in group A and group B NSTEMI: non-ST-elevation myocardial infarction

NSTEMI	Group A			Group B	
	Frequency	%		Frequency	%
No	73	43.5		67	50.8
Yes	95	56.5		65	49.2
NSTEMI	N	Mean	Standard deviation		Independent t-test (p-value)
Group A	168	0.510	0.501		0.580 (0.563)
Group B	96	0.469	0.501		

The data in Table 4 demonstrate that 44.6% of patients in group A underwent balloon angioplasty; this was higher than the proportion in group B, i.e., 22.7%, who had this procedure (p-value of <0.05).

**Table 4:** Number of balloon angioplasty procedures in group A and group B

Balloon angioplasty	Group A		Group B	
	Frequency	%	Frequency	%
No	93	55.4	102	77.3
Yes	75	44.6	30	22.7
Angioplasty	N	Mean	Standard deviation	Independent t-test (p-value)
Group A	168	0.450	0.499	4.044 (0.000)
Group B	132	0.227	0.420	

Table 5 shows the percentage of patients who underwent angioplasty with stent insertion; this was equivalent for the two groups, i.e., 85.1% of patients in group A and 87.9% of patients in group B.

**Table 5:** Number of angioplasty with stent procedures in group A and group B

Angioplasty with stent	Group A		Group B	
	Frequency	%	Frequency	%
No	25	14.9	16	12.1
Yes	143	85.1	116	87.9
Angioplasty with stent	N	Mean	Standard deviation	Independent t-test (p-value)
Group A	168	0.900	0.302	1.318 (0.189)
Group B	96	0.840	0.365	

The data presented in Table 6 show that for groups A and B, 17.9% and 15.2% of patients required readmission, respectively.

**Table 6:** Number of patient readmission in group A and group B

Readmission	Group A		Group B	
	Frequency	%	Frequency	%
No	138	82.1	112	84.8
Yes	30	17.9	20	15.2

## Discussion

The COVID-19 pandemic has placed an enormous burden on healthcare systems worldwide, which has far-reaching ramifications for medical practice. Additionally, it has resulted in changes to routine cardiac care, notably for individuals presenting with acute coronary syndromes (ACS). This study demonstrated an overall decrease in percutaneous coronary intervention (PCI) activity of 21.4% during the COVID-19 outbreak. This supports the findings of other studies performed during the pandemic and may be attributed to several factors. Another significant finding of this study was the higher rate of readmissions in group B, which may be explained by the recommendation of the Saudi MOH about reducing patients’ length of stay to minimize the risk of infection and virus transmission as the post-infective complication of myocarditis [11].

During the pandemic, there were substantial concerns regarding suitable personal protective equipment for healthcare personnel and patients staying away from healthcare institutions. Several explanations for patients’ deferrals during the pandemic can be proposed. Firstly, there was a specific patient-based apprehension relating to visiting hospitals because of COVID-19. The official government’s order to remain home may have promoted this tendency. Secondly, cardiac care could be delayed further if outpatient care capacity was depleted during the lockdown. Thirdly, the triage of patients was based on priority levels, with patients for presumed elective operations placed on a waiting list; this, therefore, deterred individuals from seeking medical assistance for chest problems quickly. With the commencement of quarantine, there was a rapid implementation of virtual clinics that resulted in an even lower number of hospital visits, including to the interventional cardiology department. Patients could also receive their medications at their own homes, as many hospitals in the Kingdom of Saudi Arabia (KSA) delivered the required drugs, thus preventing disease complications and limiting the number of patients presenting to the emergency departments.

There were several potential reasons for the observed decline in PCI procedures. The Saudi Heart Association issued guidelines for the management of patients with ACS. It was recommended that one catheterization laboratory per hospital should be active. As the pandemic progresses, it would appear that these effects are gradually decreasing [11,12].

A few other studies have already reported on changes in PCI activity during the pandemic but have yet to look at differences in either patient profiles or outcomes. Between January and March 2020, Garcia et al.’s study of nine high-volume cardiac catheterization facilities in the United States found a 38% drop in ST-elevation myocardial infarction (STEMI) activities [13]. The current research has revealed that neither the risk profile nor the clinical outcomes of individuals with STEMI varied appreciably. Similarly, Rodriguez-Leor et al. observed a decline in interventional cardiology activity. Data from 73 centers suggested a 48% overall drop in PCI and a 40% decrease in PCI procedures in cases of STEMI [14]. These results, however, were generated from a survey that did not consider specific patient features or clinical outcomes. The underlying reason for such reduced rates was that patients avoided seeking medical attention and were reluctant to visit hospitals owing to concerns regarding contracting SARS-CoV-2. Additionally, there was a shortage of intensive care unit beds as coronavirus patients mainly occupied these.

Interventional cardiology resources in the KSA are scarce; however, compared to prior to COVID-19, Daoulah et al.’s study indicated a significant decline in the incidence of STEMI in 16 hospitals throughout the KSA during the COVID-19 pandemic [15]. Kwok et al.’s study evaluated the correlation between the COVID-19 pandemic and the performance of PCI for STEMI patients in England. This study indicated a drastic reduction in the number of procedures, i.e., 43%, compared to the numbers before the pandemic [16].

According to Daoulah et al.’s study, it was found that there was no significant difference in the features of patients admitted with ACS between the two time periods. Patients’ demographics during the lockdown indicated only a minor rise in cardiovascular risk factors and comorbidities, i.e., obesity, hypertension, smoking, age over 60 years, and a history of CAD. Similarly, another study in the KSA observed no significant change in patients’ characteristics during the periods considered, i.e., before and after COVID-19, respectively [15].

The King Abdulaziz Cardiac Center (KACC) encountered no decrease in physician numbers and accepted referrals from other hospitals in Riyadh, thus showing constant activity and ongoing clinical practice throughout the pandemic. There was a lesser decline in both STEMI and non-ST-elevation myocardial infarction (NSTEMI) groups of patients compared to the previous studies and when judged against other centers in the region. The reduction in PCI activity observed in this study is consistent with the data reported in Spain and the United States [13,14].

This study has some drawbacks, most notably its small study sample and the fact that it was only a single-center study. These limitations imply that the results needed to be more sufficiently powered to demonstrate causality in the broader population. Another significant restriction was the study’s retrospective nature, with the information source limited to data reported in the healthcare system. Therefore, additional research should be conducted to address the quality and adequacy of clinical practice in other health sectors. Finally, there needs to be a clearer understanding of how local policies at each hospital may have changed due to the COVID-19 crisis, which may be driving the decline in procedures. Thus, a future study should be conducted in the region across multiple centers, which includes a larger sample size and considers differences in patient demographics. This research will help facilitate the development of guidelines for adequately managing interventional cardiology patients and allow physicians and hospitals to act accordingly during future crises. This drop in the number of procedures is attributed to several factors, one of which is people’s apprehension about visiting the emergency room despite putting their lives at greater risk, and authorities should take this into consideration when planning for the future as this might potentially propose challenges in the next pandemics.

## Conclusions

In conclusion, this retrospective review of medical records reveals a significant decrease in the number of percutaneous coronary interventions performed in the interventional cardiology department of KACC during the COVID-19 pandemic. While the incidence of acute coronary syndromes remained stable, delays in treatment due to patient fears or healthcare resource constraints may have had negative consequences for patients. The high prevalence of comorbidities such as hypertension and obesity in the patient population underscores the importance of the proactive management of these risk factors to improve outcomes. Further research is needed to fully understand the impact of COVID-19 on patients and healthcare systems, particularly in the context of cardiac care. As the pandemic continues to evolve, it is essential for healthcare providers to remain vigilant in their efforts to provide safe and effective care while minimizing the risk of COVID-19 transmission. Ultimately, the findings of this study emphasize the importance of maintaining robust healthcare systems that can adapt to crises and provide the best possible care for all patients.
